# Intrapatient Evolutionary Dynamics of Human Immunodeficiency Virus Type 1 in Individuals Undergoing Alternative Treatment Strategies with Reverse Transcriptase Inhibitors

**DOI:** 10.1089/aid.2015.0035

**Published:** 2015-07-01

**Authors:** Jonathan K. Kayondo, Nicaise Ndembi, Chris M. Parry, Patricia A. Cane, Stephane Hué, Ruth Goodall, David T. Dunn, Pontiano Kaleebu, Deenan Pillay, Jean L. Mbisa

**Affiliations:** ^1^ Uganda Virus Research Institute (UVRI), Uganda Research Unit on AIDS, Entebbe, Uganda.; ^2^ Medical Research Council (MRC)/UVRI, Uganda Research Unit on AIDS, Entebbe, Uganda.; ^3^ Virus Reference Department, Public Health England, London, United Kingdom.; ^4^ Department of Infection and Immunity, University College London, London, United Kingdom.; ^5^ MRC Clinical Trials Unit, University College London, London, United Kingdom.

## Abstract

Structured treatment interruption (STI) has been trialed as an alternative to lifelong antiretroviral therapy (ART). We retrospectively performed single genome sequencing of the HIV-1 *pol* region from three patients representing different scenarios. They were either failing on continuous therapy (CT-F), failing STI (STI-F), or suppressing on STI (STI-S). Over 460 genomes were generated from three to five different time points over a 2-year period. We found multiple-linked-resistant mutations in both treatment failures. However, the CT-F patient showed a stepwise accumulation of diverse, linked mutations whereas the STI-F patient had lineage turnover between treatment periods with recirculation of wild-type and resistant variants from reservoirs. The STI-F patient showed a 7-fold increase in the third codon position substitution rate relative to the first and second positions compared to a 2-fold increase for CT-F and increased purifying selection in the *pol* gene (62 vs. 22 sites, respectively). An understanding of intrapatient viral dynamics could guide the future direction of treatment interruption strategies.

Antiretroviral therapy (ART) for human immunodeficiency virus type 1 (HIV-1) infection is life-long. Therefore, structured treatment interruption (STI) was proposed more than a decade ago as a possible alternative management strategy to continuous treatment (CT) to reduce the cost and toxicity of ART.^1^ STI is applicable to patients in three main scenarios: (1) those initiating therapy during acute infection, (2) those with chronic drug-suppressed infection, and (3) those with advanced infection and undergoing treatment failure from multidrug-resistant virus.^2^ It is thought that STI, in those with suppressed viremia, could enhance recovery of the weakened anti-HIV immune response and therefore provide better HIV control^3^ while at the same time offering breaks off treatment to reduce side effects and cost. In contrast, in those failing treatment the benefit from interruptions could result from the reversal of the virus population to wild-type, which could potentially enhance success with subsequent salvage therapy.^4^

Comparisons of the effects of STI and CT strategies on treatment outcomes have shown conflicting results. On the one hand, studies have found evidence of patient benefit from STI with regard to certain important clinical parameters, yet in other studies STI appears to be inferior to CT. STI benefit has been most evident during acute infection when the immune system of the patient is nearly intact and where resultant expansion of virus-specific T cell immune response enables prolonged discontinuation of highly active antiretroviral therapy (HAART) with contained viral rebounds.^5,6^ One major concern though is that the viral rebound levels might still be above the threshold to prevent transmission.^7^

However, much controversy remains over the benefits of STI to successfully suppress chronically infected patients with advanced disease where HIV control during interruption critically depends on the status of the immune system. Some studies have suggested that benefits can be realized in some patients, especially if treatment is augmented to further stimulate the immune system, particularly if the STI strategy is guided by CD4^+^ T cell count monitoring.^8^ In contrast, other studies have found no benefit and associate STI with poorer clinical outcomes, particularly in terms of increased risk for opportunistic infections and death.^3,9^ Among patients with chronic HIV infection who are failing due to multidrug-resistant HIV, most studies have found that although STI results in shifts in viral populations from resistant to wild-type (drug-sensitive) virus, CD4^+^ T cell counts also decline without accompanying virological benefits to subsequent salvage therapy, as resistance quickly reemerges upon resumption of treatment.^2^

Treatment interruptions are currently not recommended as routine clinical practice, but there are genuine reasons, such as the onset of adverse events, that necessitate treatment disruptions. Therefore, elucidating viral evolution during interrupted treatment is important. Studies have concentrated on comparisons of STI with CT mostly in terms of the development of clinical events and virological, immunological, and quality of life treatment outcome.^2,6^ As yet, viral evolutionally dynamics including linkages of emerging mutations at a genome level during the course of treatment are still poorly understood.

The patterns of the emergence, reversion, decay, rebound, and polymorphism of mutations are some of the viral evolutional phenomena still confounding our knowledge of HIV-1 drug resistance. For instance, the mechanism by which resistance persists and HIV rebounds after cessation of treatment is not completely understood. One of the proposed hypotheses is that these could come from a minority resistant quasispecies in the plasma RNA and/or archived proviral DNA reservoirs, respectively. Archived reservoirs are a concern because they are assumed to be long-lived, thus making recycling of drugs toward which resistance has already emerged futile.^10^

In this study, we assessed the impact of different treatment/outcome scenarios on within host viral evolutionary dynamics. We undertook single genome sequencing of stored plasma samples from three patients within the DART trial in Uganda undergoing different treatment strategies: continuous therapy with emerging resistance (CT-F), structured treatment interruptions in a patient failing to completely suppress on ART (STI-F), and suppressing (STI-S), where viral rebound is only during the ART off-cycles.

The ART regimen was a combination of zidovudine/lamivudine/nevirapine (AZT/3TC/NVP). The STI protocol consisted of cycling on–off STI periods: 12 weeks off and 12 weeks on drugs starting from week 52 of initiating therapy (Fig. 1). Single genome sequences for the protease (PR) and reverse transcriptase (RT) regions were generated from samples at three to five time points as follows: −2, 60, 72, 108, and 120 weeks before or after initiation of therapy for both the CT-F and STI-F patients, and weeks −2, 60, and 84 for the STI-S patient. Weeks 60, 84, and 108 fell in the STI OFF cycles, whereas the rest except week −2 were ON cycles. Week −2 was a pretherapy sample (baseline). Subtyping analysis of the PR–RT regions using the REGA HIV-1 Subtyping Tool showed that all three patients were infected with HIV-1 subtype D.

**FIG. 1.**
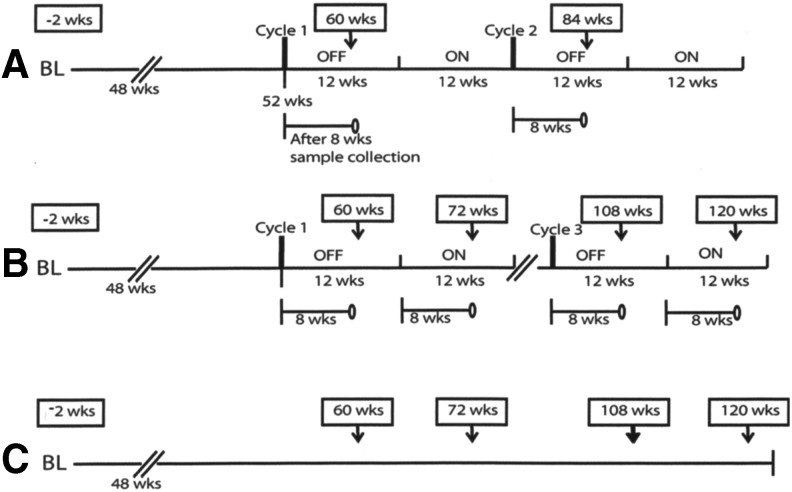
Schematic representing the treatment and sampling protocols used in this study. The three patients were each either **(A)** suppressing on structured treatment interruption (STI-S), **(B)** failing on structured treatment interruption (STI-F), or **(C)** failing on continuous therapy (CT-F). The STI protocol was cycles of 12 weeks off and 12 weeks on drugs [zidovudine/lamivudine/nevirapine (AZT/3TC/NVP)] starting from week 52 of initiating therapy. Samples used in the study were collected at different time points shown in the *boxes* on top of the schematic.

Analyses of the intrapatient trends in the emergence of drug-resistant mutations show differences between the patients. Overall, no major RTI drug-resistant mutations were detected at baseline in single genomes generated from all three patients. We found extensive, linked multidrug resistance, defined as resistance to more than one class of drug, that later emerged within both patients failing treatment (STI-F and CT-F) irrespective of therapy structure, though the evolutionary profiles differed between the patients. In the STI-F patient resistance mutations emerged during the on-treatment phase and subsequently decayed during off-treatment, whereas for the CT-F patient a gradual accumulation and retention of mutations over time were observed (Fig. 2A and B). In particular, the STI-F patient developed the following mutations by week 48: D67N/G, M184V, G190A, and M230L, with additional mutations such as K103N and N348I emerging subsequently.

**FIG. 2.**
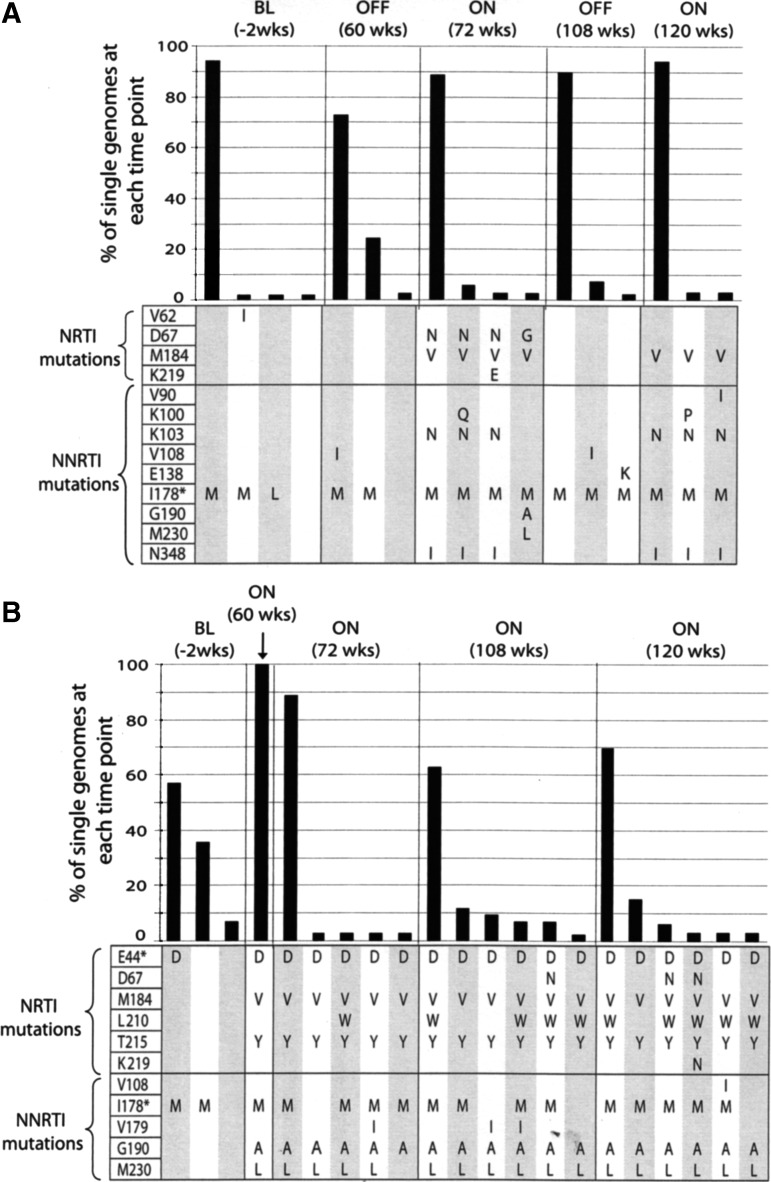
Reverse transcriptase inhibitor drug resistance-associated mutations (DRMs) in single genomes generated from patients failing therapy. **(A)** Patient failing on structured treatment interruption (STI-F) and **(B)** patient failing on continuous treatment (CT-F). The tables below each figure show linked mutations associated with resistance to nucleoside reverse transcriptase inhibitors (NRTI) and nonnucleoside reverse transcriptase inhibitors (NNRTI) on each genome and the bar graphs above depict the proportions of single genomes containing the linked mutations at each time point. *Drug resistance-associated accessory mutations linked to DRMs.

Sampling during the off-treatment period, which was 8 weeks after stopping treatment, detected no resistance mutations. Of note, not all resistant lineages reemerged in the STI-F patient when treatment was reinitiated. Lineages containing the D67N, M230L, and G190A mutations decayed while those containing the K103N, M184V, and N348I mutations reemerged. In the CT-F patient, mutations G190A, M230L, and M184V also developed early together with the T215Y mutation. However, all the resistance mutations remained stable throughout the treatment period followed by the emergence of additional mutations such as L210W. In addition, lineages containing resistance mutations were linked to the accessory mutations at position 178 (isoleucine to methionine in both patients) and/or position 44 (glutamic acid to aspartic acid in the CT-F patient). In contrast, no resistance mutations were detected in the patient suppressing under STI in the off-treatment phase.

The intrapatient evolution of the HIV lineages was further investigated using a Bayesian Markov chain Monte Carlo (MCMC) approach under strict and relaxed molecular clock models of evolution. At least two MCMC searches were carried out for sequences from each patient using the Bayesian skyline plot (BSP) demographic model and either the general time reversible (GTR) or SRD06 codon model of substitution. These were run for 50,000,000 generations with sampling every 5,000th generation after a 10% burn-in. The molecular clock model with a significant Bayes Factor (BF >20) was used.

Figure 3 shows maximum clade credibility trees of the single genomes from each patient. This shows that under the STI-F patient the viruses from ON-treatment cycles (Fig. 3A, closed symbols) were derived from a separate baseline species to that of the OFF-treatment cycles (Fig. 3A, open symbols). In contrast, the CT-F patient shows clonal expansion with all sequences generated ON-treatment deriving from a single baseline species that subsequently diverged into multiple lineages containing drug-resistant mutations (Fig. 3B, closed symbols). In addition, STI-F OFF-treatment variants had multiple origins from the baseline species, a pattern similar to that observed in the STI-S OFF-treatment variants (Fig. 3C).

**FIG. 3.**
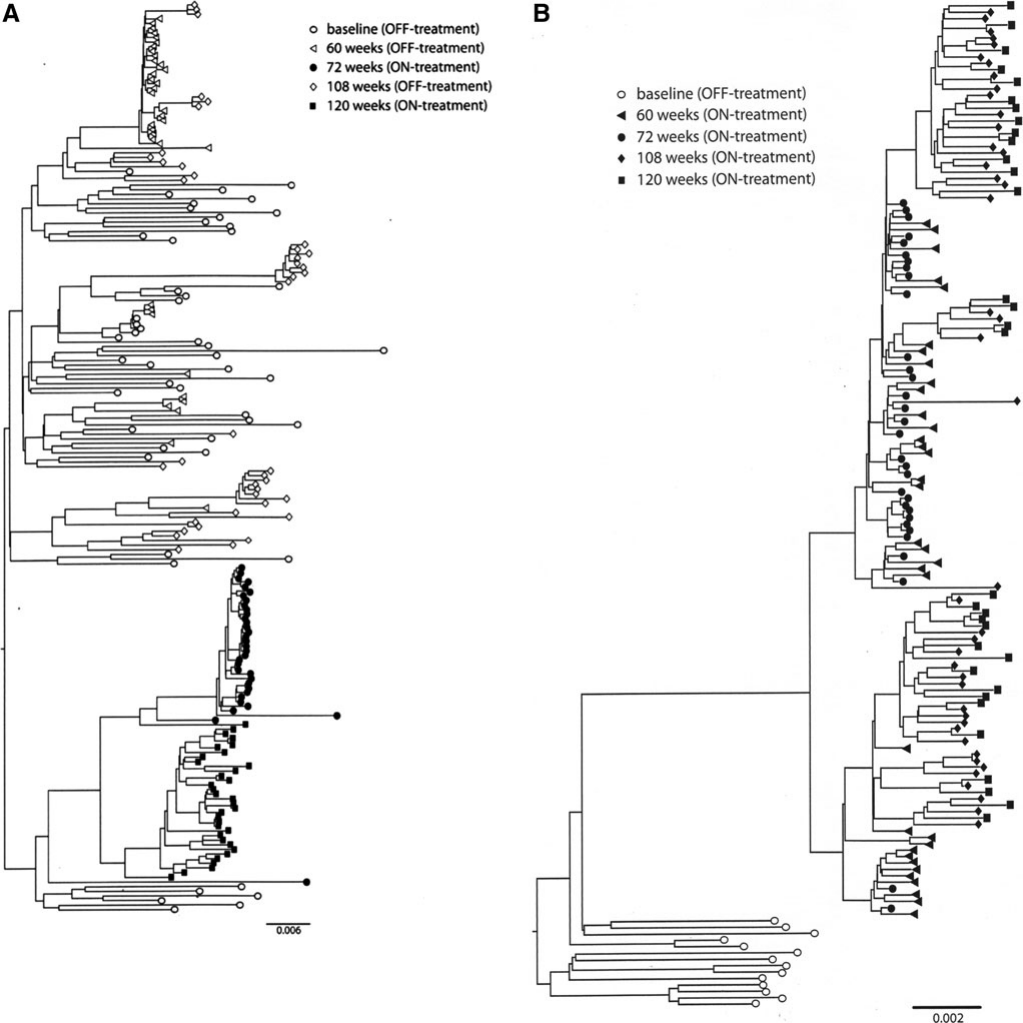

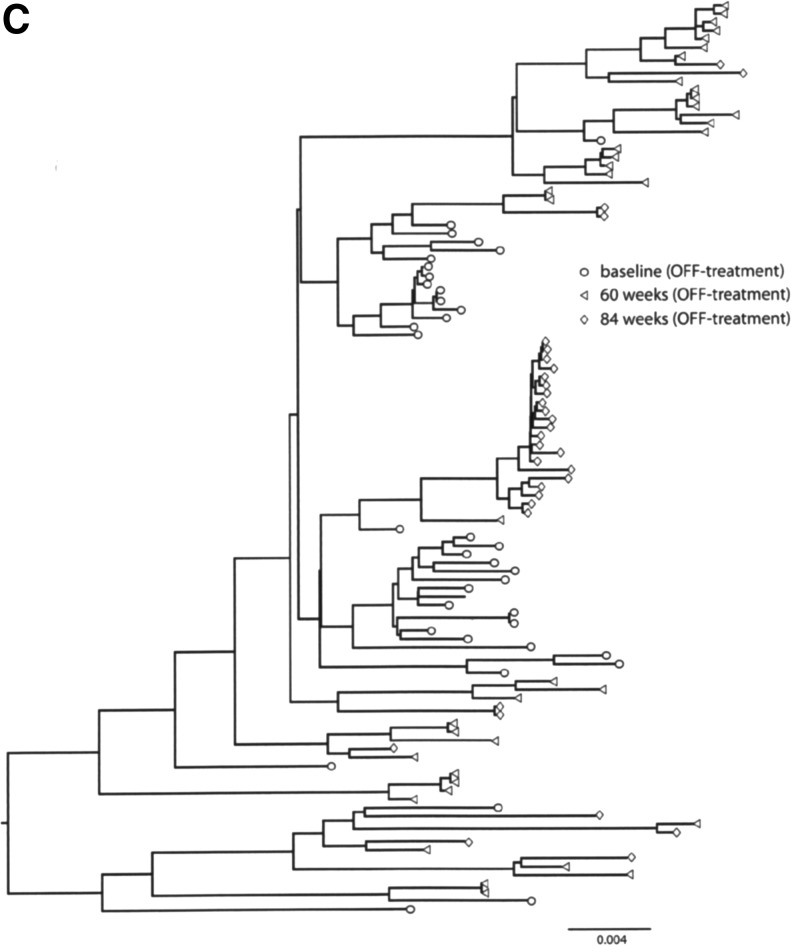
Phylogenetic reconstruction of viral lineages in the patients undergoing STI or CT. Bayesian maximum clade credibility trees (MCCT) were constructed from the single genome sequences generated from the patient on failing STI **(A)**, failing CT **(B)**, or suppressing STI **(C)**. Branches are drawn to scale with the bar and value at the bottom representing nucleotide substitutions per site. The time of sampling of each sequence is indicated by different symbols at the tips of the branches as shown in the legend next to the tree with *open symbols* representing OFF-treatment time points and *closed symbols* representing ON-treatment time points.

Examination of intrapatient viral diversity through time using Bayesian skyline plots revealed viral population bottlenecks around the time of initiating treatment in all patients, but the diversity steadily increased following treatment failure or withdrawal of therapy (Fig. 4). However, additional bottlenecks were observed in the CT-F patient during therapy, which could indicate complicated evolutionary dynamics or could represent adherence problems. Overall, viral diversity was higher in the CT-F patient, peaking toward the end of the sampling period, compared to the STI patient, where diversity decreased and remained low ON- and OFF-treatment. The analysis also revealed that the intrapatient evolutionary rates for all three treatment scenarios were similar at 2.9×10^−3^ nucleotide substitutions per site per year [2.3–3.6×10^−3^; 95% highest probability density (HPD)] in STI-F patient, 3.0×10^−3^ (2.6–3.5×10^−3^; 95% HPD) in the CT-F patient, and 1.9×10^−3^ (1.4–2.3×10^−3^; 95% HPD) in the STI-S patient.

**FIG. 4.**
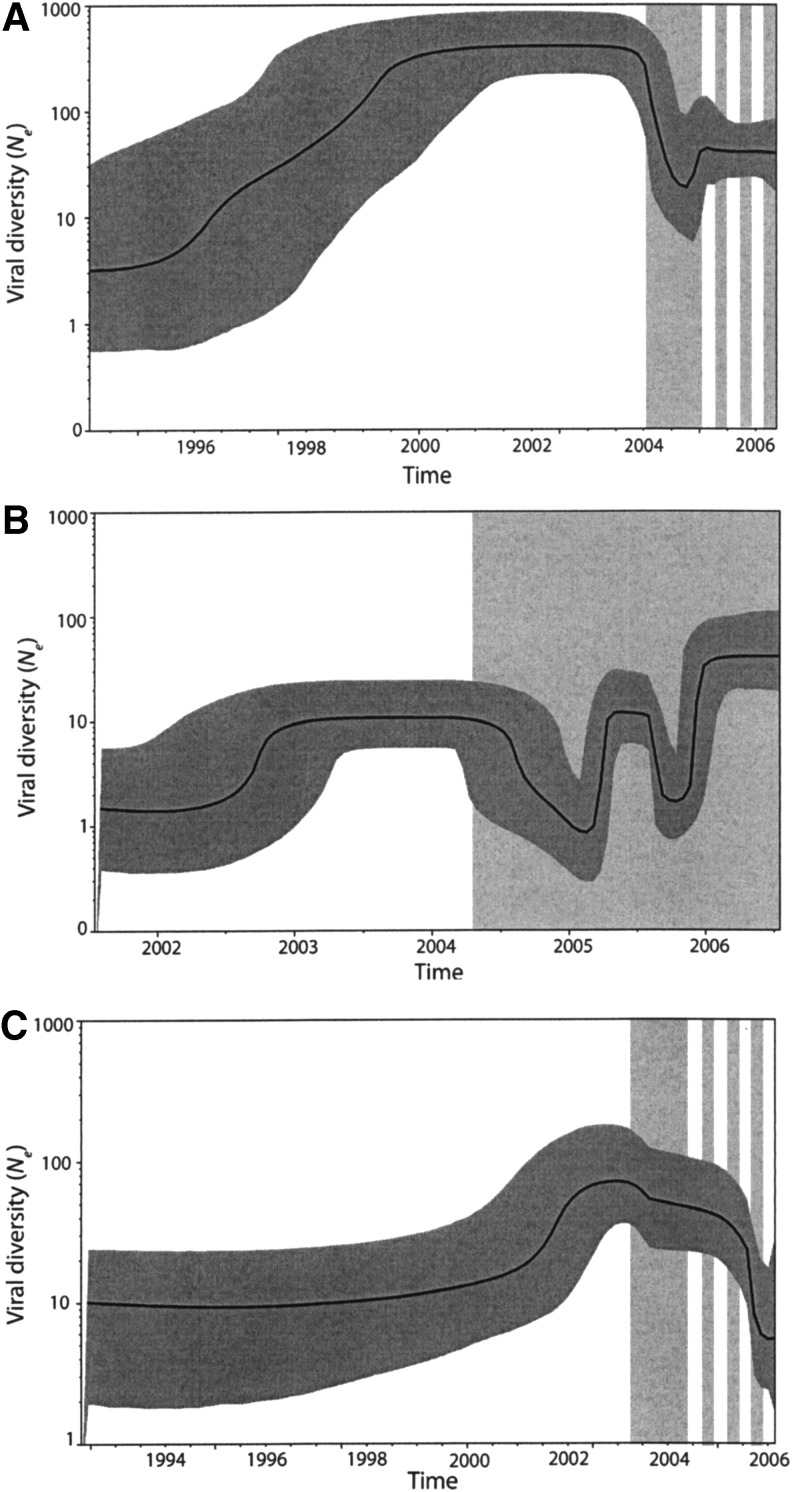
Intrapatient viral diversity during different treatment strategies. Bayesian skyline plots showing the viral diversity in the patient failing STI **(A)**, failing CT **(B)**, or suppressing on STI **(C)**. The *thick solid line* is the median estimate of viral diversity (effective population size, *N_e_*) and the *gray overlay* is the 95% highest posterior density limits. The *perpendicular regions* represent ON-treatment (*gray*) and OFF-treatment (*white*) periods.

Using the codon-based SRD06 model of substitution we show that in the STI-F patient the substitution rate was significantly higher (7-fold) at the third codon position (μ^3rd^) relative to the first and second positions (μ^1st+2nd^). In contrast, μ^3rd^ relative to μ^1st+2nd^ was 2-fold higher in the CT-F patient and intermediate in the STI-S patient at 5-fold higher. These data suggest that the selective pressure may be stronger in the STI-F patient than the CT-F patient and that this is probably dominated by purifying selection. To explore this further we estimated the site-specific selective pressures on the RT gene, the main target of the antiretrovirals in the treatment regimen used in the patients. We employed the Datamonkey web interface of the HY-PHY package, which quantifies the ratio of nonsynonymous changes to synonymous changes using three different algorithmic methods: fixed effects likelihood (FEL), single likelihood ancestor counting (SLAC), and Fast Unconstrained Bayesian AppRoximation (FUBAR), after accounting for the potentially confounding effect of recombination. Sites identified using two or more algorithms were considered significant.

This analysis showed a significant increase in the number of negatively selected sites (purifying selection) in the STI-F patient with 42 sites compared to 25 and 18 sites in the STI-S and CT-F patients, respectively (Table 1). This includes 13 drug-resistant-associated positions: K70, F77, V118, E138, Q151, L210, and P225 in STI-F; D67, V75, V106, V108, and T215 in STI-S and V62 and V106 in CT-F. In contrast, only 1, 2, and 0 positively selected sites were identified in STI-F, CT-F, and STI-S, respectively, these being V245 (STI-F) and G196 and L210 (CT-F). The relative increase in purifying selection was not limited to the RT gene as a similar level of negatively selected sites was observed in the PR gene (17, 4, and 8 in STI-F, CT-F, and STI-S, respectively).

**Table 1.** Position of Sites Under Negative Selection Across the Reverse Transcriptase Gene of Three Patients Undergoing Different Treatment Strategies and Outcomes

	*STI-F*			*STI-S*			*CT-F*		
*Codon position*	*FUBAR^a^*	*FEL*	*SLAC*	*FUBAR*	*FEL*	*SLAC*	*FUBAR*	*FEL*	*SLAC*
2	+	+							
12				+	+				
14	+						+	+	
18	+	+	+						
20	+	+	+						
21	+	+	+						
25	+	+	+						
26	+	+	+						
34	+	+	+						
37	+	+	+						
38	+			+	+				
46	+	+	+						
56	+	+	+						
**62**							+	+	
66	+	+	+	+	+				
**67**				+	+	+			
**70**	+	+	+						
**75**				+	+				
**77**	+	+	+						
86	+	+	+						
91				+	+				
92							+	+	
96				+	+				
97	+	+					+	+	+
**106**				+	+		+	+	
**108**				+	+				
112	+	+							
**118**	+	+	+						
124							+	+	
125	+	+							
126				+	+				
127	+	+							
128	+			+	+				
131	+	+	+						
134	+	+							
136				+	+	+			
**138**	+	+	+						
143							+	+	
144	+	+	+						
**151**	+	+							
157	+	+	+	+	+	+	+	+	+
163							+	+	
168				+	+	+			
169	+	+							
183							+	+	
186	+	+	+	+	+				
189	+	+	+						
192	+	+	+						
196	+	+	+						
197				+	+	+			
199	+	+	+						
201				+	+				
206	+	+	+						
208	+	+	+						
209	+	+	+	+	+				
**210**	+	+	+						
211	+	+	+						
214	+	+	+				+	+	+
**215**				+	+	+			
220	+			+	+	+			
223							+	+	
**225**	+	+	+						
228	+	+	+	+	+				
231	+	+	+				+	+	
237					+		+	+	+
241	+						+	+	+
242				+	+	+			
246	+	+	+						
247	+	+	+						
249				+	+	+	+	+	+
262				+	+	+		+	
267				+	+	+	+	+	
268	+						+	+	
276	+			+	+	+			
278				+	+	+			
280	+			+	+	+			
281	+	+							
282	+	+	+						
285	+	+	+			+			
Total		45			28			18	

^a^ Selection analysis method: fixed effects likelihood (FEL), single likelihood ancestor counting (SLAC), and Fast Unconstrained Bayesian AppRoximation (FUBAR).

*Note*: positions identified using two or more selection methods were considered significant and are shown *shaded in gray*. Drug resistant-associated mutations are shown in *bold*.

This study provided an opportunity to explore in detail the intrapatient evolutionary dynamics in patients undergoing alternative treatment strategies. The patients were part of the DART trial and were treated following the WHO guidelines with no viral loads during treatment, so those failing remained on therapy until either CD4^+^ count or clinical indicators suggested a switch to second line was needed. The data show significant differences in intrapatient viral dynamics between the treatment strategies with different pathways to the development of drug resistance.

We found that mutations conferring extensive multidrug resistance emerged regardless of treatment structure in the failing cases. The major mutations D67N/G, M184V, G190A, and M230L emerged early, whereas others such as N348I and K103N appeared later. Previous studies have reported that drug-resistant mutations persist at different rates with M184V among those showing fast reversion and with D67N and K103N less inclined to rapidly revert.^11^ M184V is known to bring about a significant reduction in viral fitness,^12^ so it would be expected to revert early, whereas K103N has little impact on replication so it can be tolerated for much longer. However, all three mutations were shown to be genetically linked and this could explain why they disappeared concomitantly within 8 weeks after interruption of treatment in the STI-F patient.

In the CT patient mutations emerge and then accumulate gradually over time, whereas in the STI entire lineages can be lost during the OFF phase not to be selected again in the subsequent ON phases as was the case for G190A, M230L, and D67G/N to some extent, though others such as M184V, N348I, and K103N reemerge in subsequent ON phases. It has been postulated that intermittent treatment, in a structured way, as opposed to continued treatment, might be beneficial as it delays resistance evolution by allowing reversion to drug-sensitive wild-type virus during the periods when drug pressure is off, thus prolonging the potency of the life-span of the drugs.

Our study shows that this might not be entirely true but could depend on the type of mutations and possibly the background sequence. The reasons why some reemerged while others did not are not clear. It could merely have been stochastic or it could depend on the background sequence and/or the accumulation of compensatory mutations. A larger sample size is required to analyze this in more detail. Lastly, drug resistance evolution was evident during the suboptimal treatment response indicating that there is pressure from the drugs that still requires viral adjustments in order to restore fitness. This confirms the notion that failing regimens might not be completely useless.^13^

Genetic diversity plays a key role in HIV-1 adaptation and drives the emergence of resistance, hence affecting the response to antiretroviral treatment.^14^ However, it is not known how treatment structure (continuous vs. interrupted) drives viral evolution in terms of diversity. We found that as expected, drug uptake results in a sharp drop in viral diversity compared to pretreatment levels in both cases followed by a gradual recovery. This finding is in line with earlier reports.^15^ Comparisons between the two treatment structures at weeks 72 and 120 (on time points in the STI) showed that CT led to the emergence of more diversified mutation profiles, with greater persistence of mutations, and the selection of TAMs by a different pathway. This was perhaps reflective of the sustained drug pressure during continuous treatment leading to a gradual and continuous evolution as opposed to a more stochastic evolution during treatment interruption. This resulted in a more diverse population in the CT-F patient, which could further aid the evolution of drug resistance.

In contrast, our data also provide additional evidence against the notion that interrupted treatment can facilitate the elimination of resistant genomes by allowing wild-type outgrowth of resistance lineages during the OFF-therapy time points. Instead, our results suggest that resistant virus fades from detection and persists either as minority variants or archived reservoirs.

The findings from this study show significant differences in viral dynamics between different treatment strategies and outcomes; however, they are from a single patient in each treatment arm, and we did not specifically investigate for ART regimen-resistant minority variants at failure. Thus, further studies are required using a larger cohort to fully understand how treatment structure might have a variably impact on host viral evolutionary dynamics. The observed decrease in viral diversity and increased purifying selection in STI patients could be an area that could be used to inform the design of better treatment interruption strategies for HIV.

## Sequence Data

The GenBank accession numbers for the patient sequences are as follows: CT-F: KP738731–KP738889, STI-F: KP738890–KP739087, and STI-S: KP739088–KP739195.

## References

[1] Lisziewicz J, *et al.*: Control of HIV despite the discontinuation of antiretroviral therapy. N Engl J Med 1999;340(21):1683–1684 10348681 10.1056/NEJM199905273402114

[2] Pai NP and Klein MB: Structured treatment interruptions in chronic HIV management: Where next? Expert Rev Anti Infect Ther 2006;4(6):909–912 17181404 10.1586/14787210.4.6.909

[3] Oxenius A, *et al.*: Stimulation of HIV-specific cellular immunity by structured treatment interruption fails to enhance viral control in chronic HIV infection. Proc Natl Acad Sci USA 2002;99(21):13747–13752 12370434 10.1073/pnas.202372199 PMC129766

[4] Hirschel B: Planned interruptions of anti-HIV treatment. Lancet Infect Dis 2001;1(1):53–59 11871414 10.1016/S1473-3099(01)00022-6

[5] Goujard C, *et al.*: Continuous versus intermittent treatment strategies during primary HIV-1 infection: The randomized ANRS INTERPRIM Trial. AIDS 2012;26(15):1895–1905 22842994 10.1097/QAD.0b013e32835844d9

[6] Weber J, Porter K, and Babiker A: Short-course antiretroviral therapy in primary HIV infection. N Engl J Med 2013;368(21):2036–2037 23697518 10.1056/NEJMc1303486

[7] Hamlyn E, *et al.*: Plasma HIV viral rebound following protocol-indicated cessation of ART commenced in primary and chronic HIV infection. PLoS One 2012;7(8):e43754 22952756 10.1371/journal.pone.0043754 PMC3432055

[8] Maggiolo F, *et al.*: CD4 cell-guided scheduled treatment interruptions in HIV-infected patients with sustained immunologic response to HAART. AIDS 2009;23(7):799–807 19114869 10.1097/QAD.0b013e328321b75e

[9] Lundgren JD, *et al.*: Inferior clinical outcome of the CD4+ cell count-guided antiretroviral treatment interruption strategy in the SMART study: Role of CD4+ cell counts and HIV RNA levels during follow-up. J Infect Dis 2008;197(8):1145–1155 18476293 10.1086/529523

[10] Turriziani O, Andreoni M, and Antonelli G: Resistant viral variants in cellular reservoirs of human immunodeficiency virus infection. Clin Microbiol Infect 2010;16(10):1518–1524 20673260 10.1111/j.1469-0691.2010.03329.x

[11] Booth CL, *et al.*: Prevalence and predictors of antiretroviral drug resistance in newly diagnosed HIV-1 infection. J Antimicrob Chemother 2007;59(3):517–524 17213262 10.1093/jac/dkl501

[12] Nicastri E, *et al.*: Replication capacity, biological phenotype, and drug resistance of HIV strains isolated from patients failing antiretroviral therapy. J Med Virol 2003;69(1):1–6 12436471 10.1002/jmv.10269

[13] Deeks SG, *et al.*: Interruption of treatment with individual therapeutic drug classes in adults with multidrug-resistant HIV-1 infection. J Infect Dis 2005;192(9):1537–1544 16206068 10.1086/496892

[14] Hemelaar J, *et al.*: Global trends in molecular epidemiology of HIV-1 during 2000–2007. AIDS 2011;25(5):679–689 21297424 10.1097/QAD.0b013e328342ff93 PMC3755761

[15] Joos B, *et al.*: HIV rebounds from latently infected cells, rather than from continuing low-level replication. Proc Natl Acad Sci USA 2008;105(43):16725–16730 18936487 10.1073/pnas.0804192105 PMC2575487

